# Supplementary material for: Development and crystal structures of a potent second-generation dual degrader of BCL-2 and BCL-xL
Source: Nat Commun. 2024 Mar 29;15:2743. doi: 10.1038/s41467-024-46922-4 (PMC10979003; doi:10.1038/s41467-024-46922-4)
Supplement: Supplementary file 1 — Supplementary Information [file 41467_2024_46922_MOESM1_ESM.pdf]

**Supplementary Information**

# *Development and crystal structures of a more potent second-generation dual degrader of BCL-2 and BCL-xL*

Digant Nayak[1,4], Dongwen Lv[1,4], Yaxia Yuan[1], Peiyi Zhang[2], Wanyi Hu[2], Anindita Nayak[1], Eliza A. Ruben[1], Zongyang Lv[1], Patrick Sung[1], Robert Hromas[3], Guangrong Zheng[2*], Daohong Zhou[1*], and Shaun K. Olsen[1*]

[1] Department of Biochemistry & Structural Biology and Greehey Children's Cancer Research Institute, University of Texas Health Science Center at San Antonio, San Antonio, TX, 78229, USA

[2] Department of Medicinal Chemistry, College of Pharmacy, University of Florida, Gainesville, FL, 32610, USA

[3] Department of Medicine, University of Texas Health Science Center at San Antonio, San Antonio, TX, 78229, USA

[4] These authors contributed equally to this work

*Correspondence should be addressed to: zhengg@cop.ufl.edu; zhoud@uthscsa.edu; olsens@uthscsa.edu

**Keywords:** PROTAC, ubiquitin, E3 ligase, ternary complex, BCL-xL/BCL-2, crystal structure, degrader

**Supplementary Information Inventory:**

Supplementary Table 1-2

Supplementary Figures 1-14

**Supplementary Table 1 | Crystallographic Data and Refinement Statistics**

| | VCB/753b/BCL-xL complex | VCB/753b/BCL-2 complex | VCB/WH244/BCL-2 complex |
|---|---|---|---|
| PDB ID | 8FY0 | 8FY1 | 8FY2 |
| Source | APS 24 IDE | APS 24 IDE | APS 24 IDE |
| Wavelength (Å) | 0.979 | 0.979 | 0.979 |
| Resolution Limits (Å) | 170-2.94 (3.12-2.94) | 94.6-2.56 (2.68-2.56) | 167.5-2.98 (3.14-2.98) |
| Space Group | $P2_12_12_1$ | $P2_1$ | $P2_12_12_1$ |
| Unit Cell (Å) *a*, *b*, *c* | 47.6, 118.9, 170.1 | 47.5, 94.6, 81.2 | 47.3, 102.6, 167.4 |
| Unit Cell (°) $\alpha$, $\beta$, $\gamma$ | 90.0, 90.0, 90.0 | 90.0, 97.5, 90.0 | 90.0, 90.0, 90.0 |
| Number of observations | 115599 | 157673 | 143191 |
| Number of reflections | 21196 (3282) | 22772 (2722) | 17444 (2476) |
| Completeness (%) | 99.3 (97.1) | 99.1 (96.3) | 100.0 (100.0) |
| Mean I/σI | 7.6 (1.4) | 12.2 (1.5) | 11.5 (1.1) |
| $CC_{1/2}$ | 0.99 (0.60) | 0.99 (0.80) | 0.99 (0.50) |
| $R_{merge}$ [a] | 0.175 (0.999) | 0.137 (0.953) | 0.199 (2.21) |
| $R_{pim}$ | 0.121 (0.698) | 0.085 (0.590) | 0.107 (1.15) |
| | | | |
| Refinement Statistics | | | |
| Resolution Limits (Å) | 69.2-2.94 (3.02-2.94) | 80.5-2.56 (2.63-2.56) | 83.7-2.98 (3.07-2.98) |
| # of reflections (work/free) | 21130/1998 | 22689/1997 | 17054/1707 |
| Completeness (%) | 99.2 (93.0) | 98.8 (93.0) | 99.2 (93.0) |
| # atoms (protein/ligand/solvent) | 3909/112/12 | 3915/112/-- | 3928/115/-- |
| $R_{cryst}$ [b] | 0.198 (0.290) | 0.195 (0.291) | 0.234 (0.444) |
| $R_{free}$ | 0.242 (0.322) | 0.249 (0.349) | 0.289 (0.495) |
| Bonds (Å)/ Angles (°) | 0.004/0.950 | 0.007/0.975 | 0.010/1.11 |
| B-factors: protein/ligand/solvent (Å$^2$) | 65.4/54.9/80.0 | 66.3/60.7/-- | 92.3/81.3/-- |
| Ramachandran statistics (%) | | | |
| favored | 96.6 | 95.1 | 93.0 |
| allowed | 3.4 | 4.7 | 6.6 |
| outliers | 0.0 | 0.2 | 0.4 |
| Clashscore | 3.8- 100$^{th}$ percentile (N=90, 2.94 Å ± 0.25 Å) | 6.2- 99$^{th}$ percentile (N=226, 2.56 Å ± 0.25 Å) | 12.0- 97$^{th}$ percentile (N=74, 2.98 Å ± 0.25 Å) |

Parentheses indicate statistics for the high-resolution data bin for x-ray data.
a. $R_{merge} = \sum_{hkl} \sum_i |I(hkl)_i - \langle I(hkl)\rangle| / \sum_{hkl} \sum_i \langle I(hkl)_i\rangle$.
b. $R_{cryst} = \sum_{hkl} |Fo(hkl) - Fc(hkl)| / \sum_{hkl} |Fo(hkl)|$, where *Fo* and *Fc* are observed and calculated structure factors, respectively.

## Supplementary Table 2 | List of primers

BCL-2 E136A

F: TTTGCCACCGTGGTTGAAGCGCTGTTTCGTGATGGCGTG
R: CACGCCATACGAAACAGCGCTTCAACCACGGTGGCAAA

BCL-2 T132D

F: GCCCGTGGCCGTTTTGCCGATGTGGTTGAAGAACTGTTT
R: AAACAGTTCTTCAACCACATCGGCAAAACGGCCACGGGC

BCL-2 T132S

F: GCCCGTGGCCGTTTTGCCAGCGTGGTTGAAGAACTGTTT
R: AACAGTTCTTCAACCACGCTGGCAAAACGGCCACGGGC

BCL-2 F124L

F: CAGCTGCATCTGACCCCCTGACCGCCCGTGGCCGTTTT
R: AAAACGGCCACGGGCGGTCAGCGGGGTCAGATGCAGCTG

BCL-2 F124A

F: CAGCTGCATCTGACCCCGGCGACCGCCCGTGGCCGTTTT
R: AAAACGGCCACGGGCGGTCGCCGGGGTCAGATGCAGCTG

BCL-2 G128R

F: ACCCCGTTTACCGCCCGTCGTCGTTTTGCCACCGTGGTT
R: AACCACGGTGGCAAAACGACGACGGGCGGTAAACGGGGT

BCL-2 T125D

F: CTGCATCTGACCCCGTTTGATGCCCGTGGCCGTTTTGCC
R: GGCAAAACGGCCACGGGCATCAAACGGGGTCAGATGCAG

BCL-2 T125S

F: CTGCATCTGACCCCGTTTAGCGCCCGTGGCCGTTTTGCC
R: GGCAAAACGGCCACGGGCGCTAAACGGGGTCAGATGCAG

BCL-xL R103A

F: TTTGAACTGCGCTATCGCGCGGCATTTTCAGATTTGACC
R: GGTCAAATCTGAAAATGCCGCGCGATAGCGCAGTTCAAA

BCL-xL D107A

F: TATCGCCGCGCATTTTCAGCGTTGACCAGCCAGCTGCAT
R: ATGCAGCTGGCTGGTCAACGCTGAAAATGCGCGGCGATA

BCL-xL D107K

F: TATCGCCGCGCATTTTCAAAATTGACCAGCCAGCTGCAT
R: ATGCAGCTGGCTGGCATTTTGAAAATGCGCGGCGATA

BCL-xL S110A

F: GCATTTTCAGATTTGACCGCGCAGCTGCATATTACCCCT
R: AGGGGTAATATGCAGCTGCGCGGTCAAATCTGAAAATGC

BCL-xL Q111A

F: TTTTCAGATTTGACCAGCGCGCTGCATATTACCCCTGGT
R: ACCAGGGGTAATATGCAGCGCGCTGGTCAAATCTGAAAA

BCL-xL Q111K

F: TTTTCAGATTTGACCAGCAAACTGCATATTACCCCTGGT
R: ACCAGGGGTAATATGCAGTTTGCTGGTCAAATCTGAAAA

## List of guide RNAs

**Gene Name**: BCL2L1 (BCL-xL)

**gRNA**: CTCTGAGACATTTTTATAAT

**ssODN**:
GAGAATCACTAACCAGAGACGAGACTCAGTGAGTGAGCAGGTGTTTTGGACAATGGACTGGTTGAG
CCCATCCCTATTATAAAAATGGTGAGCGGCTGGCGGCTGTTCAAGAAGATTAGCTCTCAGAGCAACC
GGGAGCTGGTGGTTGACTTTCTCTCCTACAAGCTTTCCCAGAAAGGATACAGCTGGAGTCAGTTT

**Gene Name**: BCL2 (BCL-2)

**gRNA**:TGGGAAGGATGGCGCACGCT

**ssODN**:
CTTTCTCTGGGGGCCGTGGGGTGGGAGCTGGGGCGAGAGGTGCCGTTGGCCCCCGTTGCTTTTCC
TCTGGGAAGGATGGTGAGCGGCTGGCGGCTGTTCAAGAAGATTAGCGCGCACGCTGGGAGAACAG
GGTACGATAACCGGGAGATAGTGATGAAGTACATCCATTATAAGCTGTCGCAGAGGGGCTACGAG

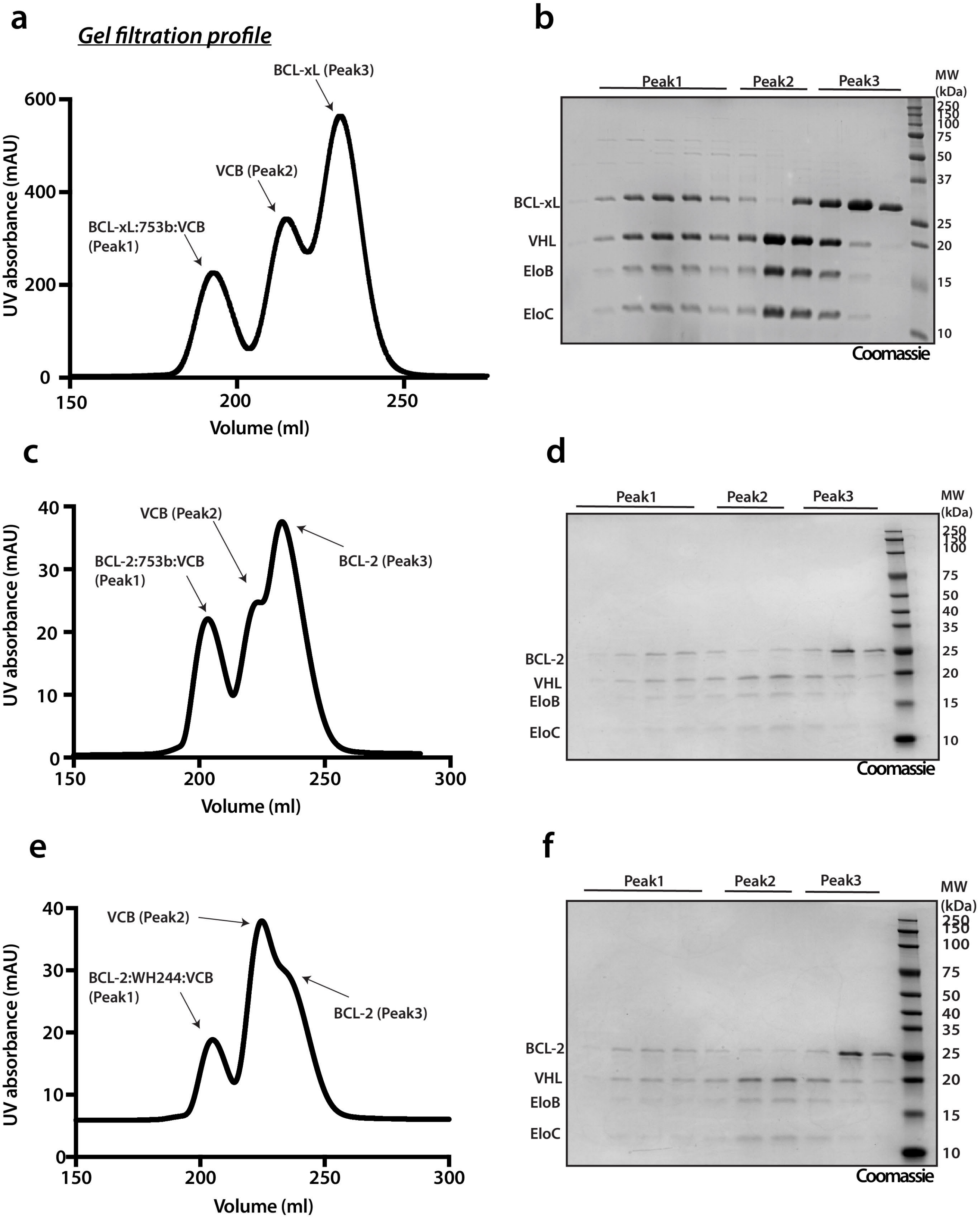


**Supplementary Fig.1 | In-solution purification of ternary complexes.** Shown here is the S200 26/600 gel filtration profile for VCB/753b/BCL-xL **a**, and the corresponding SDS-PAGE gel analysis of the peak fractions, **b**. Similarly the gel filtration and PAGE gel profiles for VCB/753b/BCL-2, **c** and **d**; and VCB/WH244/BCL-2, **e** and **f**, respectively. Peak 1 from each of the gel filtration profiles was pooled and taken for crystallization trials.

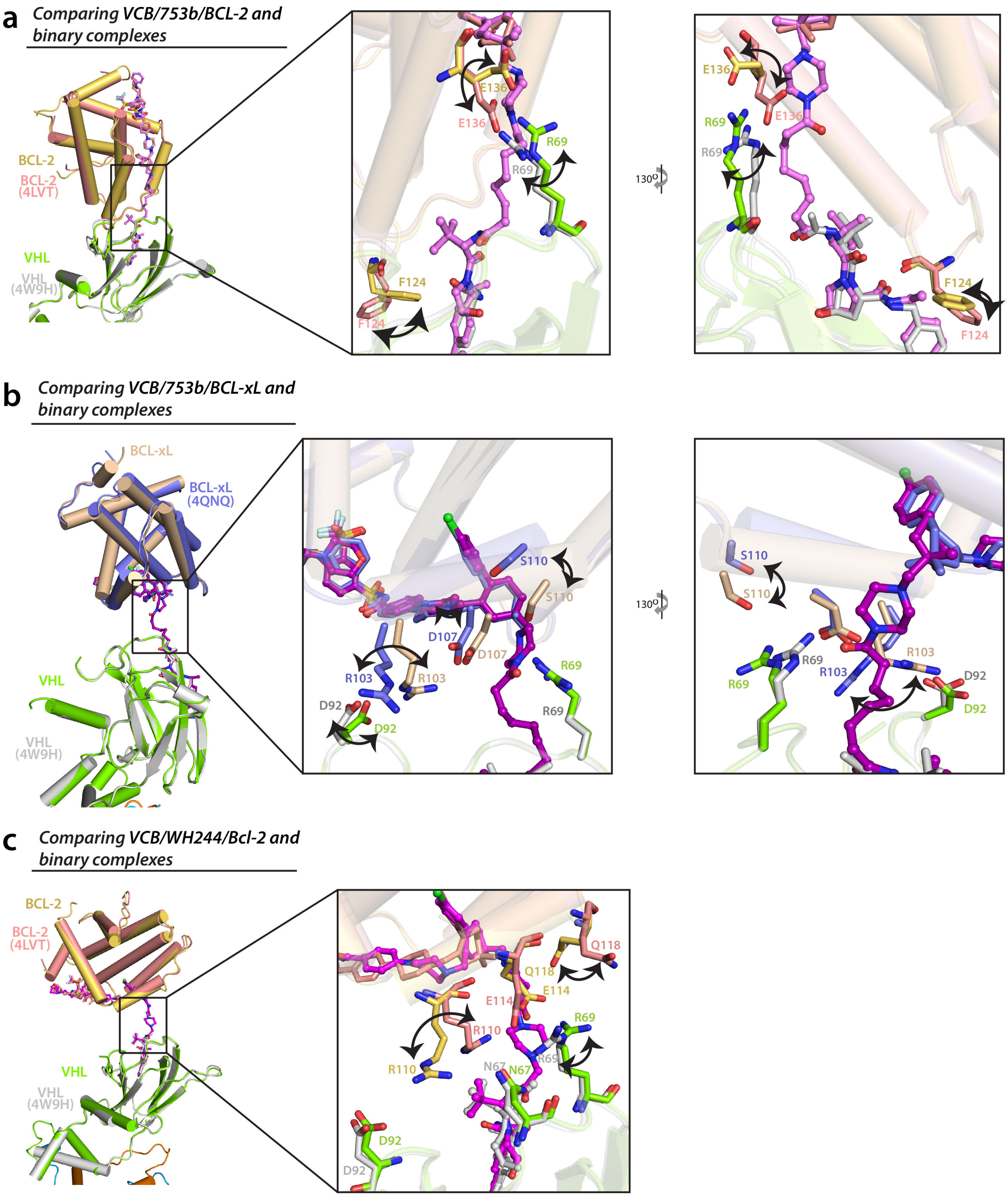


**Supplementary Fig.2 | Overlay of BCL-xL/BCL-2 and VHL in the ternary complexes with the corresponding binary complexes to show shift in residues.** **a,** Shown here is the overlay of VCB/753b/BCL-2 with VHL/ligand (PDB code 4W9H) and BCL-2/ ABT263 (PDB code 4LVT) binary complexes. The shifted residues are labeled and shown with the two-head arrows. **b,** Similarly for VCB/753b/BCL-xL and comparison with BCL-xL/ABT263 (PDB code 4QNQ) binary complex. **c,** Shown for VCB/WH244/BCL-2.

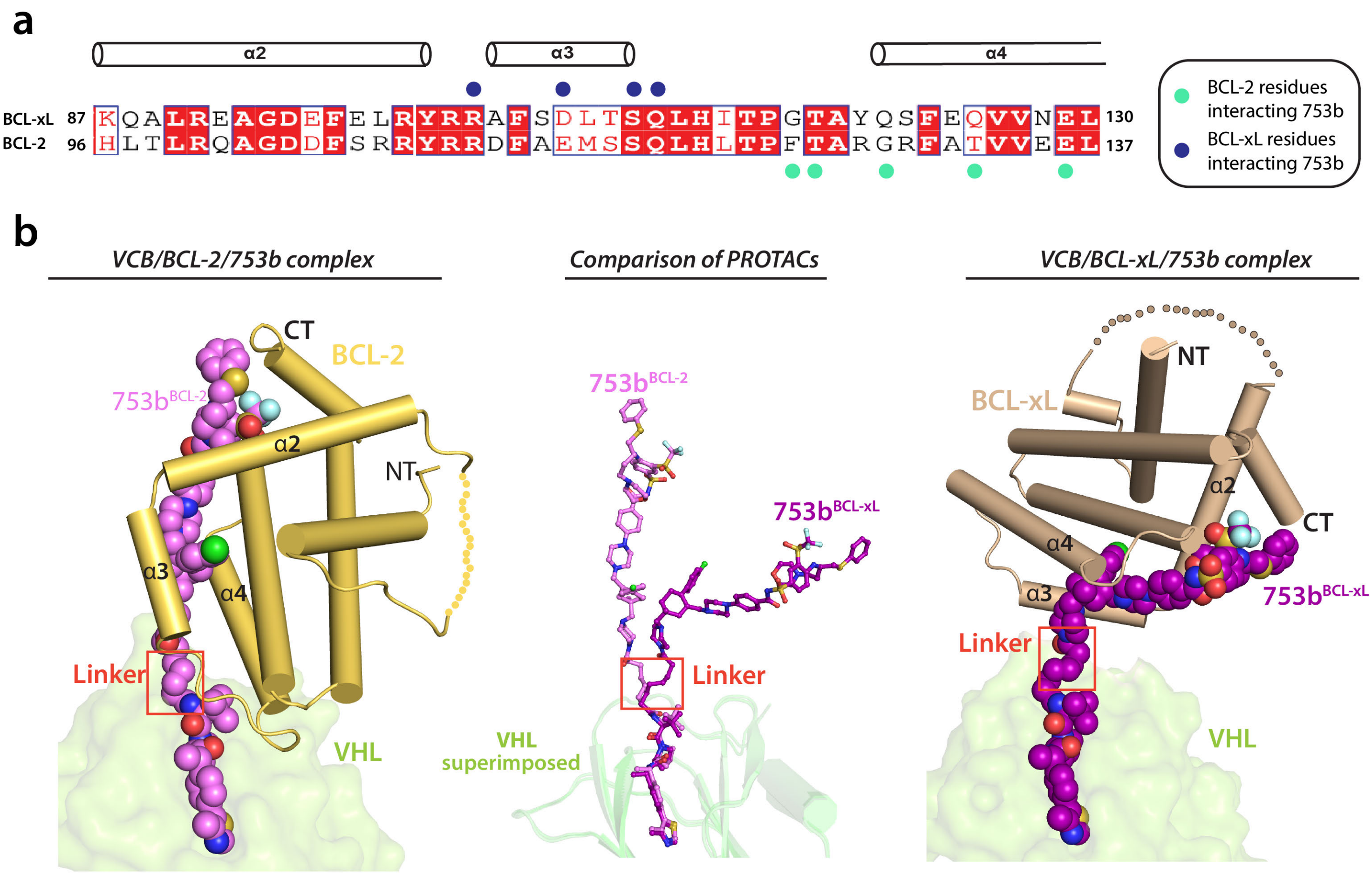


**Supplementary Fig.3 | Differences between 753b-mediated ternary structures with BCL-2 and BCL-xL.**
**a**, Structure-based sequence alignment of 753b binding regions in BCL-2 and BCL-xL. For the aligned sequences, red boxes highlight identical residues, while boxes with residues in red color indicate similar residues Secondary structure for BCL-2 is shown above. Spheres on top of the residues highlight residues propagating interactions with 753b and VHL. **b,** *Left*, BCL-2 shown as cartoon with 753b as spheres for VCB/753b/BCL-2 complex. The loops are connected through spheres and the linker of 753b is shown as red box and labeled. *Right*, Similar orientation as left for BCL-xL in VCB/753b/BCL-xL complex. *Centre*, The difference in orientation of 753b (BCL-xL and BCL-2) when both the ternary complexes are aligned via VHL. NT: N-terminal, CT: C-terminal, α1:Helix 1.

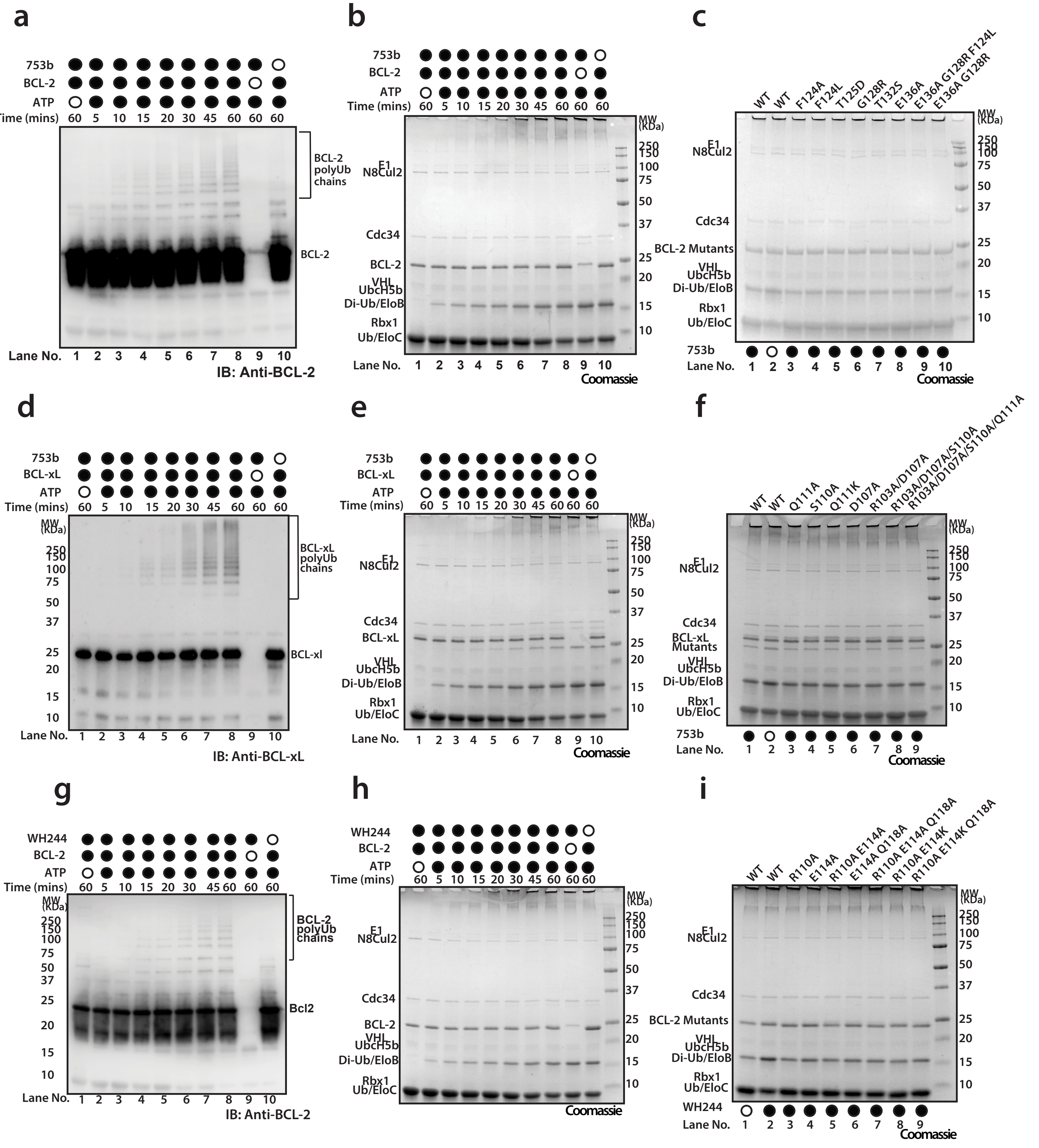


**Supplementary Fig.4 | Assay optimization for assessing the effect of mutation on ternary complex formation through immunoblotting.** **a,** Immunoblot of polyUb chain formation on WT BCL-2 in the presence or absence of 753b, BCL-2 and ATP, respectively at different time-points as indicated. **b,** The corresponding SDS-PAGE gel for the immunoblot in *a*. **c,** The SDS-PAGE for the corresponding immunoblot showing the effect of various mutants of BCL-2 used in *Fig. 2b*. Similarly for WT, mutants of BCL-xL and 753b shown in **d, e** and **f**. Similarly for WT, mutants of BCL-2 and WH244 shown in **g, h** and **i**.

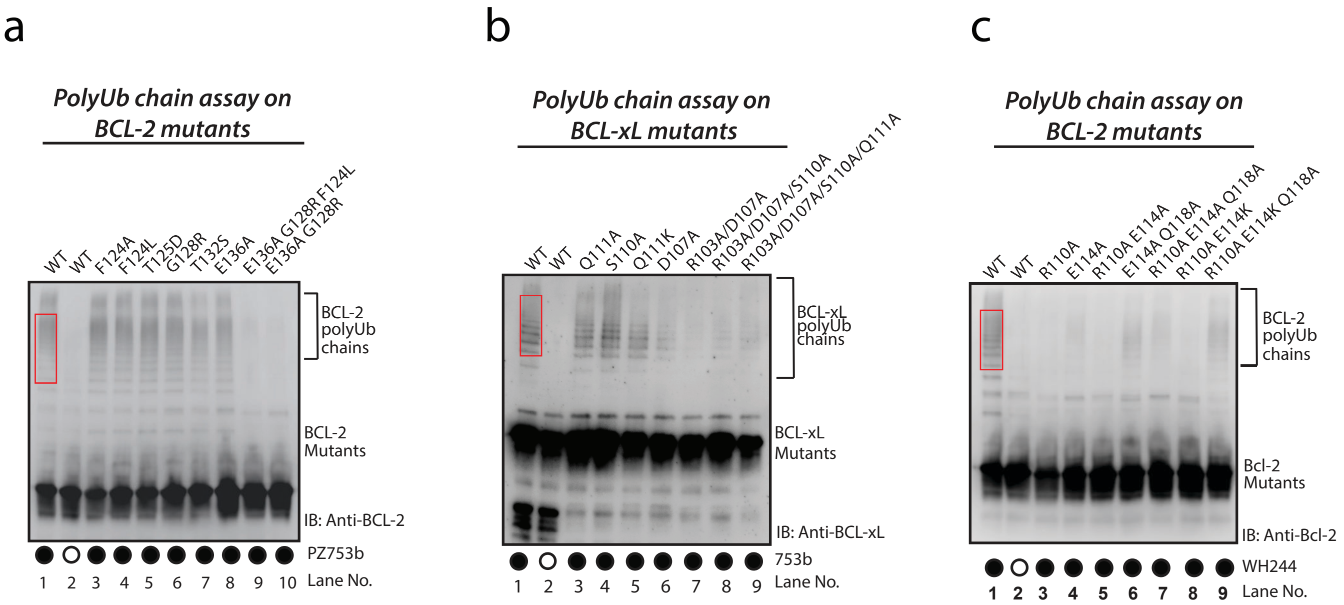


**Supplementary Fig 5 | Polyubiquitination assays to assess interfacial BCL-xL and BCL-2 mutants a,b,c.** Shown here is the western blot of polyubiqutin chain formation assay using the indicated mutants of BCL-2 with 753b (**a, Fig.2b**), BCL-xL with 753b (**b, Fig.2e**) and BCL-2 with WH244 (**c, Fig. 5d**), respectively. The data (area under the red box) was quantified presented as bar graphs in Fig2b, Fig.2e and Fig.5d, respectively. The source data are provided as source file.

**a** ***Melting curves for mutants of BCL-2 mutants***

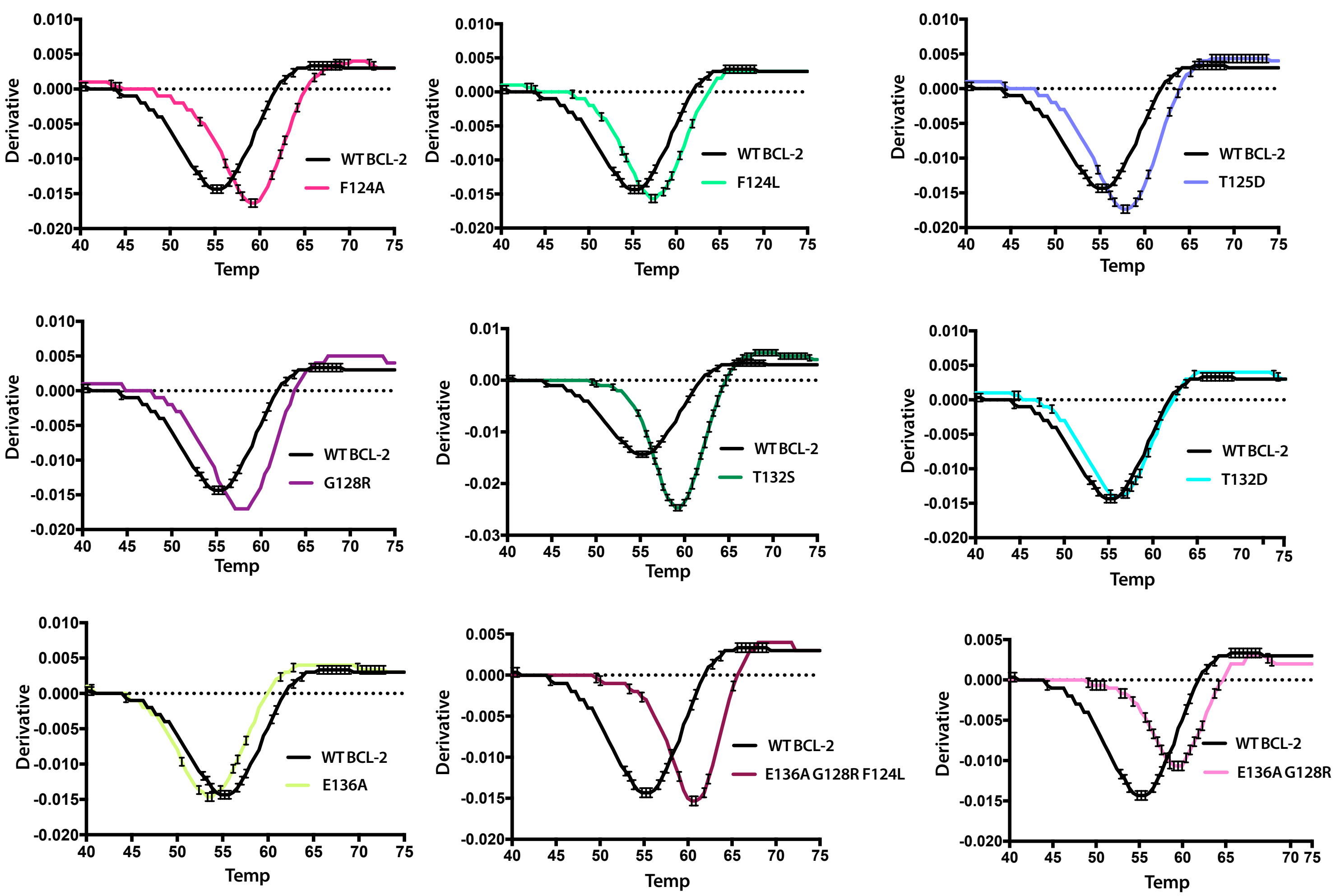


**Supplementary Fig. 6 | Effects of mutation on stability of BCL-2. a,** Data showing the melting temperature of BCL-2 and mutant proteins. A graph is plotted for derivative Vs temperature for each BCL-2 mutant separately along with the WT for each plot to compare the change in melting temperatures. The data presented are representative results from the n=3 technical replicates done independently with similar results.

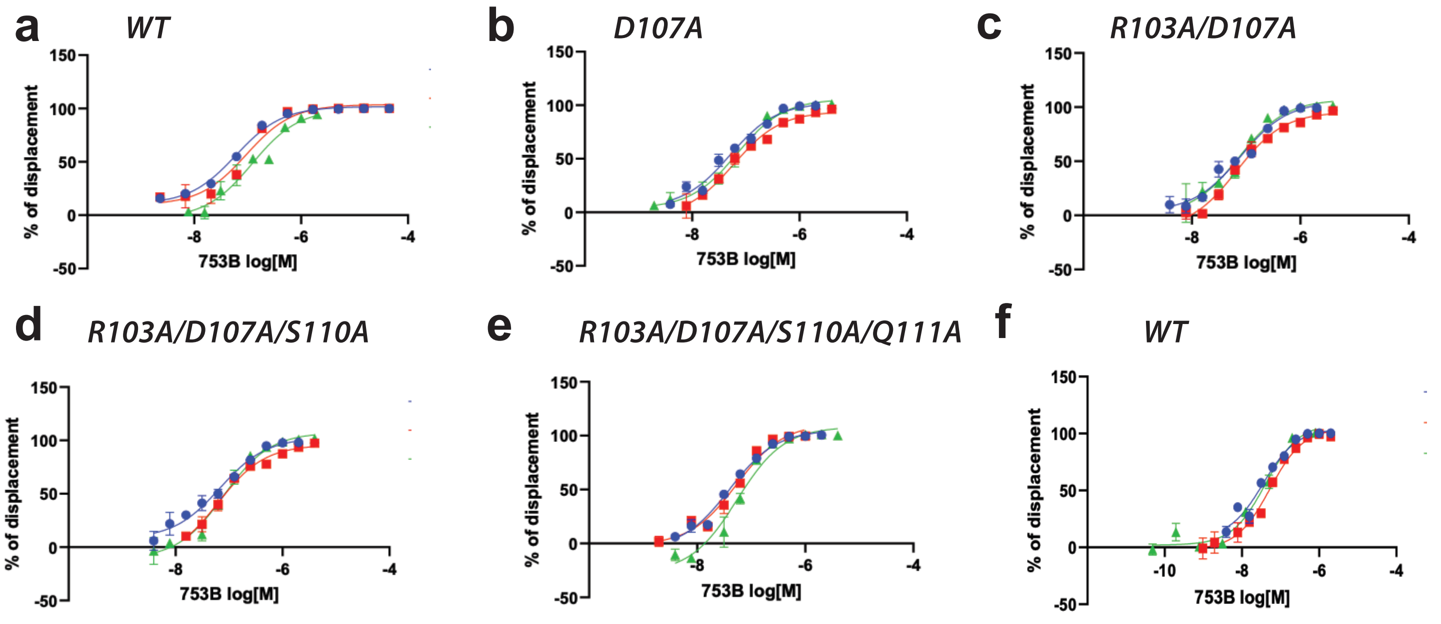


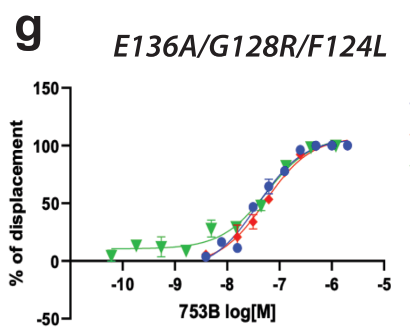


**h**

| BCL-xL Mutants | 753b`s Ki (nM, n=3) |
|---|---|
| WT | 21.9 ± 7.3 |
| D107A | 30.3 ± 4.5 |
| R103A/D107A | 36.7 ± 3.0 |
| R103A/D107A/S110A | 18.4 ± 2.3 |
| R103A/D107A/ S110A/Q111AA | 23.0 ± 4.0 |
| **BCL-2 Mutants** | **753b`s Ki (nM, n=3)** |
| WT | 24.9 ± 5.4 |
| E136A/G128R/F124L | 35.6 ± 6.9 |

**Supplementary Fig 7 | Assessing binary binding of various BCL-xl and BCL-2 mutants with 753b**
Mutant BCL-xL and BCL-2 binary binding affinity with 753b respectively, determined by AlphaScreen assay. **a-e**, Data obtained from a typical AlphaScreen assay is depicted for both wild-type (WT) and mutant BCL-2. Each graph shows three curves which represents three replicates. **f-g**, AlphaScreen assay for WT and mutant BCL-2. **h**, Ki values (in nM) represented in a tabular format for graphs a-g, as the mean ± s.d. of experiments performed in triplicates (n=3).

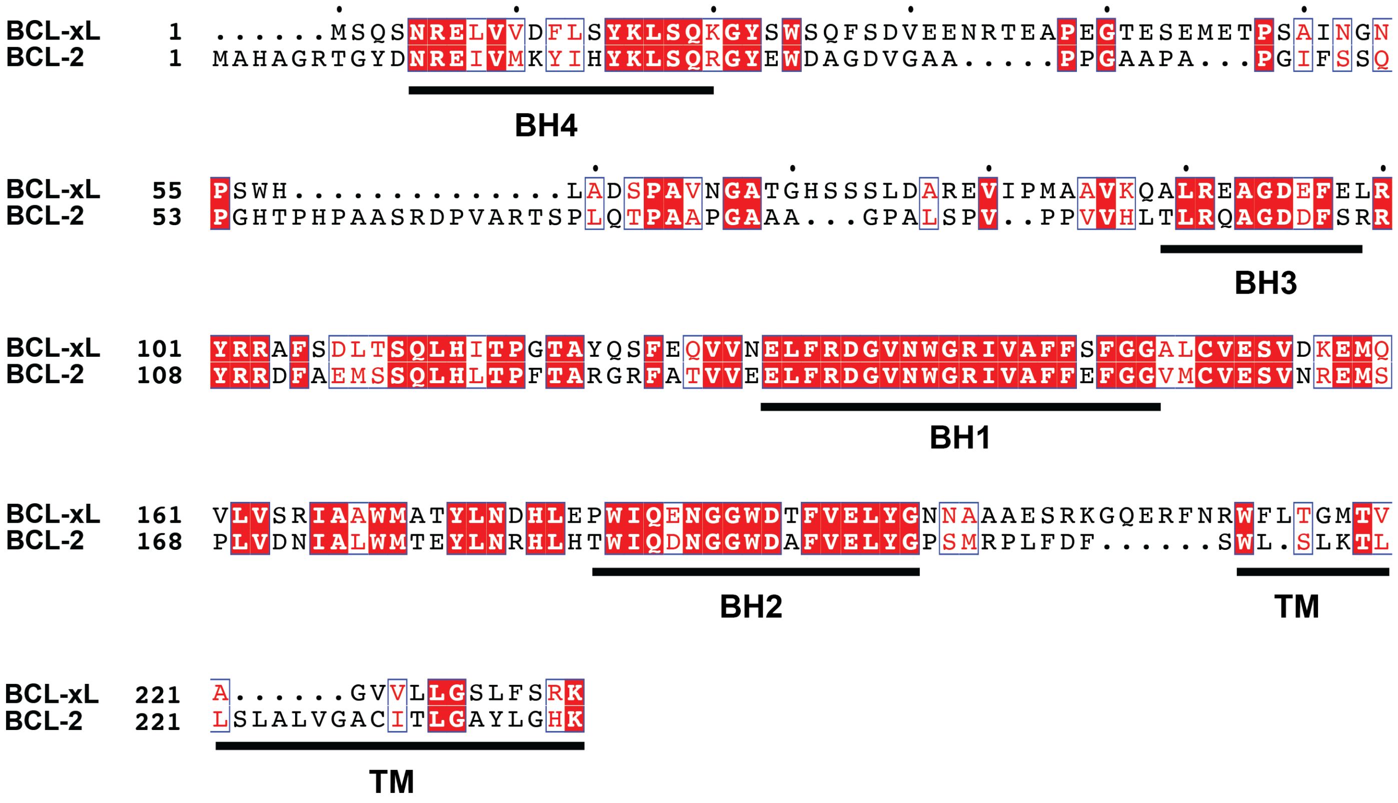


**Supplementary Figure 8 | Sequence alignment of BCL-xL and BCL-2 proteins.** BCL-2 homolgy (BH) regions present are indicated with thick black line below the sequence and labeled. For the aligned sequences, red boxes highlight identical residues, while boxes with residues in red color indicate similar residues. For all *in vitro* experiments, Transmembrane region (TM) was removed in this study. Dot indicates gap in alignment.

**a** ***Melting curves for mutants of BCL-xL mutants***

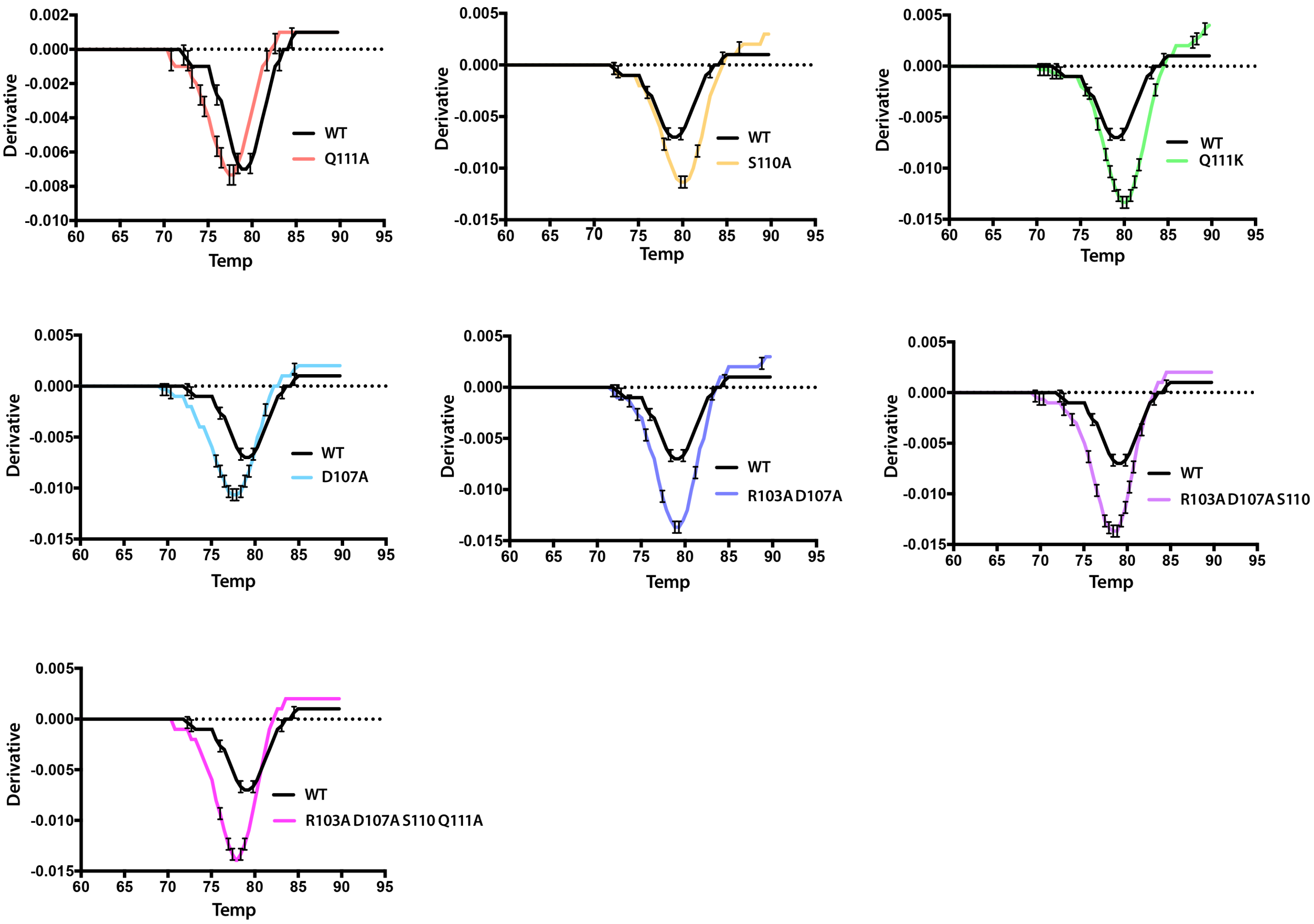


**Supplementary Fig.9 | Effects of mutation on stability of BCL-xL. a,** Data showing the melting temperature of BCL-xL and mutant proteins. A graph is plotted for derivative Vs temperature for each BCL-xL mutant separately along with the WT for each plot to compare the change in melting temperatures. The data presented are representative results from the n=3 technical replicates done independently with similar results.

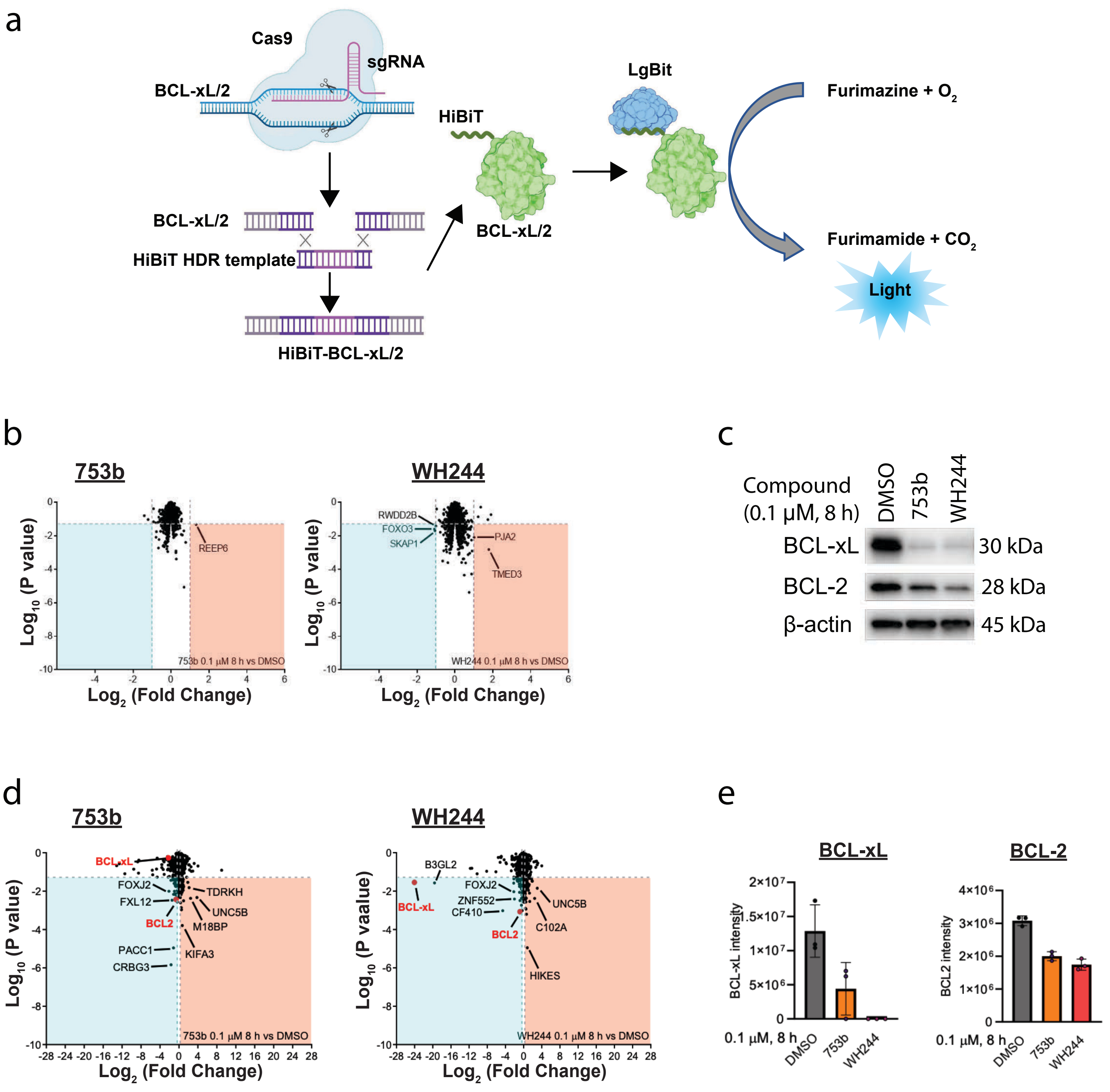


**Supplementary Fig. 10 | Schematic of HiBiT knock-in, TMT proteomics and DIA proteomics a,** Graphical representation of the generation of endogenous HiBiT knock-in BCL-xL/2 Hela cells through CRISPR-Cas9 and the HiBiT degradation assay. **b,** Effects of 753b (*left*) and WH244 (*right*) on the proteome of Jurkat cells using TMT approach**. c,** Western blot using the sample used in **b** to confirm degradation of BCL-xL/BCL-2 in a 753b and WH244 dependent manner, respectively. **d,** Effects of 753b (left) and WH244 (right) on the proteome of Hela cells using DIA approach. **e,** The raw intensity levels of BCL-xL (left) and BCL-2 (right) under the treatments of DMSO, 753b, and WH244.

a

*Binary binding assay for WT BCL-2 and compounds*

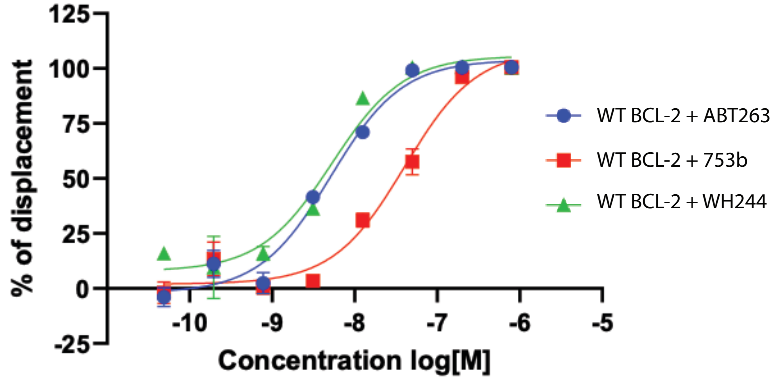


| Compounds | Ki (nM) |
|---|---|
| ABT263 | 3.0 |
| 753b | 23.4 |
| WH244 | 3.1 |

b

*Binary binding assay for WT BCL-xL and compounds*

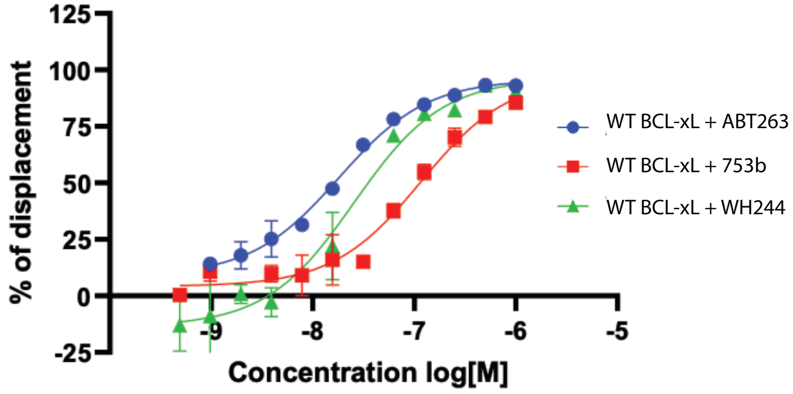


| Compounds | Ki (nM) |
|---|---|
| ABT263 | 4.4 |
| 753b | 28.0 |
| WH244 | 6.1 |

**Supplementary Fig 11 | Binary binding assay for BCL-2 and compounds.** Comparing the binary binding affinities of 753b and WH244 for BCL-2 (**a**) and BCL-xL (**b**), respectively.

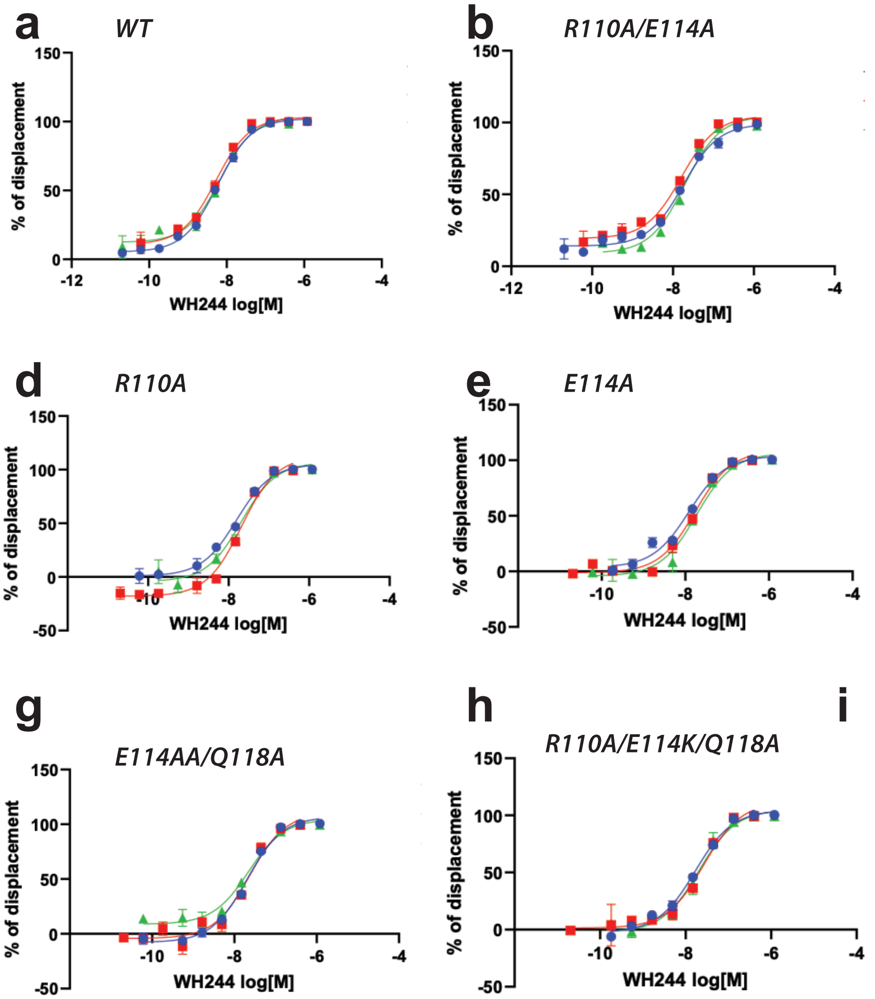


i

| BCL-2 Mutants | WH244`s Ki (nM, n=3) |
|---|---|
| WT | 3.5 ± 0.5 |
| R110A E114A | 5.8 ± 0.6 |
| R110A E114K Q118A | 7.2 ± 0.6 |
| R110A | 8.0 ± 1.2 |
| E114A | 7.4 ± 1.5 |
| R110A E114K | 7.0 ± 0.9 |
| E114A Q118A | 9.6 ± 0.7 |
| R110A E114K Q118A | 6.0 ± 1.3 |

**Supplementary Fig 12 | Assessing binary binding of various BCL-2 mutants with WH244** WT and mutant BCL-2 binary binding affinity with WH244, determined by AlphaScreen assay. **a-h**, Data obtained from a typical AlphaScreen assay is depicted for both wild-type (WT) and mutant BCL-2. Each graph shows three curves which represents three replicates. **i,** Ki values (in nM) are presented in the table for graphs a-h, as the mean ± s.d. of experiments performed in triplicates (n=3).

a

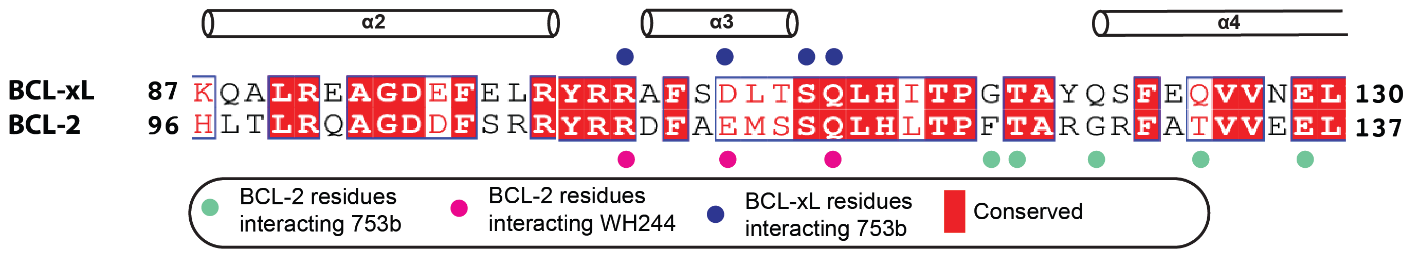


**Supplementary Fig 13 | Structure-based sequence alignment of PROTAC binding regions of BCL-2 and BCL-xL**, Secondary structure for BCL-2 is shown above the sequence. For the aligned sequences, red boxes highlight identical residues, while boxes with residues in red color indicate similar residues. Residues involved in interactions with 753b (BCL-2; F124, T125, G128, T132 and E136. BCL-xL; R103, D107, S110 and Q111.) and WH244 (BCL-2; R110, E114 and Q118) are highlighted by spheres as indicatd in the legend below the sequence.

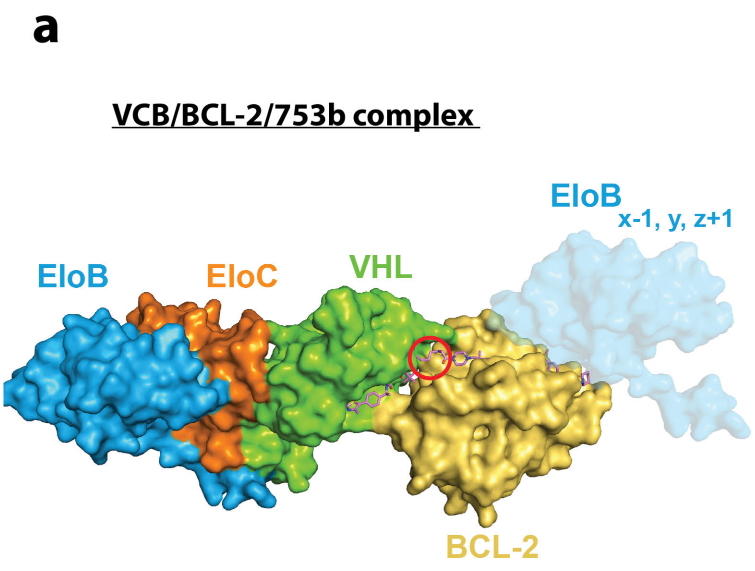


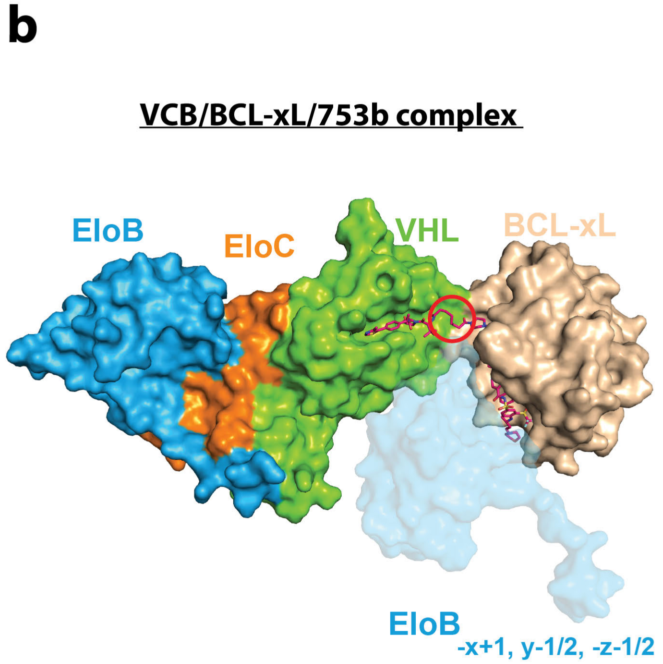


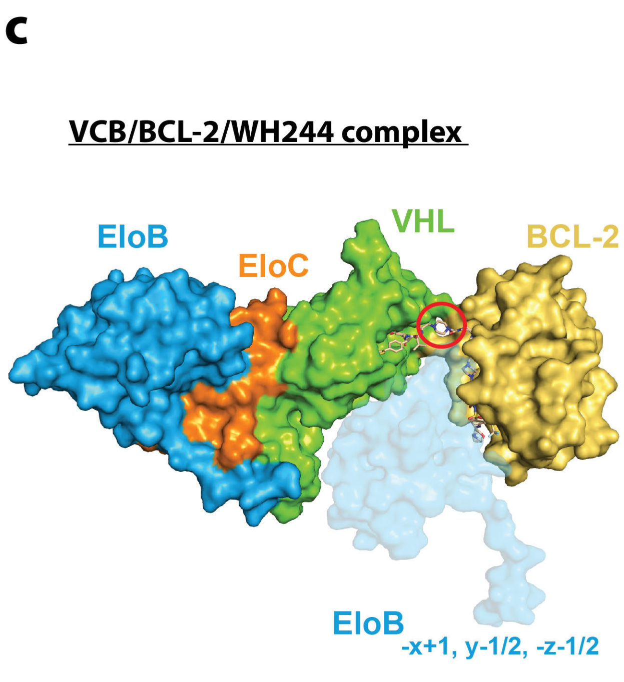


**d**

| Complex | Sym operator for EloB | BSA with Protac (Å$^2$) |
|---|---|---|
| VCB/BCL-2/753b complex | x-1, y, z+1 | 203.5 |
| VCB/BCL-xL/753b complex | -x+1, y-1/2, -z-1/2 | 239.7 |
| VCB/BCL-2/WH244 complex | -x+1, y-1/2, -z-1/2 | 327.4 |

**Supplementary Fig 14 | Influence of crystal packing in our ternary structures**. Shown here is the surface representation of crystallographically related EloB (transparent surface) copy at the interface of ternary complex formed by VCB/BCL-2/753b **(a)**, VCB/BCL-xL/753b **(b)** and VCB/BCL-2/WH244 **(c)**, respectively. The symmetery operator used to generate EloB copies is labeled at the bottom of each EloB shown as transparent surface. **d.** The buried surface area for each of the symmetery related EloB with the corresponding PROTAC molecule shown in a tabular format. The red circle denotes the linker position.
