# Supplementary material for: Development and crystal structures of a potent second-generation dual degrader of BCL-2 and BCL-xL
Source: Nat Commun. 2024 Mar 29;15:2743. doi: 10.1038/s41467-024-46922-4 (PMC10979003; doi:10.1038/s41467-024-46922-4)
Supplement: Supplementary file 2 — Reporting Summary [file 41467_2024_46922_MOESM2_ESM.pdf]

nature portfolio

Corresponding author(s): Shaun K Olsen, Daohong Zhou, Guangrong Zheng

Last updated by author(s): Feb 28, 2024

Reporting Summary

Nature Portfolio wishes to improve the reproducibility of the work that we publish. This form provides structure for consistency and transparency in reporting. For further information on Nature Portfolio policies, see our Editorial Policies and the Editorial Policy Checklist.

Statistics

For all statistical analyses, confirm that the following items are present in the figure legend, table legend, main text, or Methods section.

n/a	Confirmed	
☐	☒	The exact sample size (*n*) for each experimental group/condition, given as a discrete number and unit of measurement
☐	☒	A statement on whether measurements were taken from distinct samples or whether the same sample was measured repeatedly
☐	☒	The statistical test(s) used AND whether they are one- or two-sided<br>*Only common tests should be described solely by name; describe more complex techniques in the Methods section.*
☒	☐	A description of all covariates tested
☒	☐	A description of any assumptions or corrections, such as tests of normality and adjustment for multiple comparisons
☐	☒	A full description of the statistical parameters including central tendency (e.g. means) or other basic estimates (e.g. regression coefficient) AND variation (e.g. standard deviation) or associated estimates of uncertainty (e.g. confidence intervals)
☐	☒	For null hypothesis testing, the test statistic (e.g. *F*, *t*, *r*) with confidence intervals, effect sizes, degrees of freedom and *P* value noted<br>*Give P values as exact values whenever suitable.*
☒	☐	For Bayesian analysis, information on the choice of priors and Markov chain Monte Carlo settings
☒	☐	For hierarchical and complex designs, identification of the appropriate level for tests and full reporting of outcomes
☒	☐	Estimates of effect sizes (e.g. Cohen's *d*, Pearson's *r*), indicating how they were calculated

Our web collection on statistics for biologists contains articles on many of the points above.

Software and code

Policy information about availability of computer code

Data collection	Image Lab Touch version 2.2.0.08 Software (Bio-rad, Hercules, CA, USA) was used for scanning all immunoblots on the ChemiDoc MP imaging system; Gen5 version 3.04 software (BioTek, Winooski, VT, USA) was used for absorbance and luminescence measurements on Synergy Neo2 multi-mode plate reader.<br><br>Crystallography data collection was conducted remotely at NE-CAT beam line 24-ID-E at the Advanced Photon Source. The beam line/data collection were controlled using their standard in-house program.
Data analysis	GraphPad Prism v7 or v9 (GraphPad Software, La Jolla, CA, USA) was used for the preparation of all the graphs, determination of half maximal effective concentration (EC50) values.<br><br>Crystallography data analysis was conducted using the PHENIX v1.20.1, and COOT 0.91 software. Polyubiquitination assay data analysis was conducted using ImageJ 1.53 and GraphPad Prism v7. Thermal Shift data was analyzed by GraphPad Prism v7.

For manuscripts utilizing custom algorithms or software that are central to the research but not yet described in published literature, software must be made available to editors and reviewers. We strongly encourage code deposition in a community repository (e.g. GitHub). See the Nature Portfolio guidelines for submitting code & software for further information.

Data

Policy information about availability of data

All manuscripts must include a data availability statement. This statement should provide the following information, where applicable:
- Accession codes, unique identifiers, or web links for publicly available datasets
- A description of any restrictions on data availability
- For clinical datasets or third party data, please ensure that the statement adheres to our policy

Atomic coordinates and structure factors for VCB/753b/BCL-xL, VCB/753b/BCL-2 and VCB/WH244/BCL-2 have been deposited in the Protein Data Bank (PDB) under accession number 8FY0, 8FY1 and 8FY2, respectively.
Proteomics data for DIA-MS has been submitted in MASSIVE database (http://massive.ucsd.edu) with following database identifier: MSV000094144
Proteimics data for TMT-MS has been submitted in PRIDE database (https://www.ebi.ac.uk/pride/) with the following database identifier: PXD049976
The source data underlying Fig. 2b, d and Fig.5d are provided as a Source Data file.
Accession codes of other (published) data that was used for comparison: PDB: 4LVT, 4W9H and 4QNQ.

Human research participants

Policy information about studies involving human research participants and Sex and Gender in Research.

Reporting on sex and gender	N/A
Population characteristics	N/A
Recruitment	N/A
Ethics oversight	N/A

Note that full information on the approval of the study protocol must also be provided in the manuscript.

Field-specific reporting

Please select the one below that is the best fit for your research. If you are not sure, read the appropriate sections before making your selection.

☒ Life sciences ☐ Behavioural & social sciences ☐ Ecological, evolutionary & environmental sciences

For a reference copy of the document with all sections, see nature.com/documents/nr-reporting-summary-flat.pdf

Life sciences study design

All studies must disclose on these points even when the disclosure is negative.

Sample size	Statistical methods were not used to determine sample sizes.<br>However, for quantification purposes, a sample size of n = 2 or 3 unless otherwise stated (reproduced two or more independent times) was used to assess reproducibility and robustness of each experiment performed. Sample sizes were based on prior study in the field (For example: Alabi S, Jaime-Figueroa S, Yao Z, et al. Mutant-selective degradation by BRAF-targeting PROTACs[J]. Nature communications, 2021, 12(1): 1-11.).
Data exclusions	In general, no data were excluded from the analyses.
Replication	To ensure reproducibility of experimental findings, all biochemical or cellular assays were repeated independently at least 2-3 times. One representative result for each experiment is presented in the main Figures or the Extended Data Figures. We confirm that all attempts of replication were successful.
Randomization	Cultured cells were passaged evenly (random distribution) into dishes or flasks for treatment with PROTACs and/or indicated compounds. Due to dose-response nature of the experiments performed, randomization of the samples post-treatment would make data interpretation impossible.
Blinding	Investigators were not blinded to the nature of their samples during data collection and analysis based on prior study in the field (for example: Alabi S, Jaime-Figueroa S, Yao Z, et al. Mutant-selective degradation by BRAF-targeting PROTACs[J]. Nature communications, 2021, 12(1): 1-11.).

Reporting for specific materials, systems and methods

We require information from authors about some types of materials, experimental systems and methods used in many studies. Here, indicate whether each material, system or method listed is relevant to your study. If you are not sure if a list item applies to your research, read the appropriate section before selecting a response.

Materials & experimental systems

n/a	Involved in the study
☐	☒ Antibodies
☐	☒ Eukaryotic cell lines
☒	☐ Palaeontology and archaeology
☒	☐ Animals and other organisms
☒	☐ Clinical data
☒	☐ Dual use research of concern

Methods

n/a	Involved in the study
☒	☐ ChIP-seq
☒	☐ Flow cytometry
☒	☐ MRI-based neuroimaging

Antibodies

Antibodies used

For cell based studies, antibodies were purchased from Cell Signaling Technologies (CST) and the dilutions are as follows: BCL-xL (Cat No. 2762S, 1:1000), BCL-2 (Cat No. 2870S, 1:1000). β-actin antibody was purchased from MP Biomedicals (Cat No. 8691001, 1:20 000).
For in vitro polyubiqutination assay, following antibodies were purchased from SANTA CRUZ BIOTECHNOLOGY, INC. and the dilutions are as follows: BCL-xL (Cat. No. sc-8392, 1:1000) and BCL-2 (Cat. No. sc-7382, 1:1000).

Validation

All the used antibodies are commercially available. The antibodies used in a specific species or application have been validated by manufacturers to be used in that species/application and this information is provided in their website and/or antibody datasheets.
CST Antibody Validation Principles
To ensure our antibodies will work in your experiment, we adhere to the Hallmarks of Antibody Validation™, six complementary strategies that can be used to determine the functionality, specificity, and sensitivity of an antibody in any given assay. CST adapted the work by Uhlen, et. al., ("A Proposal for Validation of Antibodies." Nature Methods (2016)) to build the Hallmarks of Antibody Validation, based on our decades of experience as an antibody manufacturer and our dedication to reproducible science.
CST Hallmarks of Antibody Validation
We guarantee that our antibodies are fit for purpose by carefully tailoring the combination of validation strategies applied to each product. This means customizing our validation process according to the biological role of the target, while considering the sensitivity requirements of the downstream assay, the availability of appropriate testing models, and the relevance of each method to target investigation.
1. Binary Model: Antibody signal is measured in model systems with known presence/absence of target signal. Includes wild-type vs. genetic knockout, targeted induction or silencing.
2. Ranged Expression: Antibody signal strength is measured in cell lines or tissues representing a known continuum of target expression levels. Includes siRNA and heterozygous knockout assays.
3. Orthogonal Data: Antibody signal is correlated to target expression in model systems measured using antibody independent assays. Includes mass spectrometry and in situ hybridization.
4. Multiple Antibodies: Antibody signal is compared to the signal observed using antibodies targeting nonoverlapping epitopes of the target. Includes IP, ChIP, and ChIP-seq.
5. Heterologous Expression: Antibody signal is evaluated in cell lines following heterologous expression of native (or mutated) target protein.
6. Complementary Assays: Antibody specificity may be validated using complementary assays. Includes competitive ELISA, peptide dot blots, peptide blocking, or protein arrays.
The following antibodies from CST were used in this study.
BCL-xL (Cat No. 2762S, 1:1000)
https://www.cellsignal.com/products/primary-antibodies/bcl-xl-antibody/2762
BCL-2 (Cat No. 2870S, 1:1000)
https://www.cellsignal.com/products/primary-antibodies/bcl-2-50e3-rabbit-mab/2870
Antibody from MP Biomedicals (β-actin, Cat No. 8691001, 1:20 000).
Validated in our previous publication (https://doi.org/10.1038/s41591-019-0668-z)
Antibody from SANTA CRUZ BIOTECHNOLOGY, INC (BCL-xL (Cat. No. sc-8392, 1:1000) and BCL-2 (Cat. No. sc-7382, 1:1000))
Validated in previous publications (https://www.pnas.org/doi/10.1073/pnas.97.1.466 and https://journals.plos.org/plosone/article?id=10.1371/journal.pone.0139657 respectively)

Eukaryotic cell lines

Policy information about cell lines and Sex and Gender in Research

Cell line source(s)

Jurkat (Cat. No. TIB-152) and Hela (Cat. No. CCL-2) cells were recently purchased from American Type Culture Collection (ATCC, Manassas, VA, USA).

Authentication

The cell lines have been validated by the suppliers. The cell lines purchased from ATCC were purchased as 'Certified Reference Material' stocks. ATCC authenticates cell lines using morphology, karyotyping and STR profiling

Mycoplasma contamination

Cell lines were recently purchased from ATCC and were not further tested for mycoplasma contamination in our laboratory,

but cell proliferation rate and morphology of all the cell lines were continuously monitored.

Commonly misidentified lines<br>(See ICLAC register)	none
